# Nutritional Status and Cardiometabolic Risk Factors in Institutionalized Adults with Cerebral Palsy

**DOI:** 10.3390/medicina55050157

**Published:** 2019-05-17

**Authors:** Aurora Norte, Coral Alonso, José Miguel Martínez-Sanz, Ana Gutierrez-Hervas, Isabel Sospedra

**Affiliations:** 1 Nursing Department, Faculty of Health Science, University of Alicante, 03690 Alicante, Spain; aurora.norte@ua.es (A.N.); coralalonsogomez@gmail.com (C.A.); josemiguel.ms@ua.es (J.M.M.-S.); isospedra@ua.es (I.S.); 2 Research Group on Food and Nutrition (ALINUT), University of Alicante, 03690 Alicante, Spain

**Keywords:** cerebral palsy, metabolic syndrome, nutritional status, cardiometabolic risk factors

## Abstract

*Background and Objectives:* Cerebral palsy (CP) is a set of permanent disorders that limit physical activity and increase the risk of developing other diseases, such as metabolic syndrome (MS). Adequate nutrition can contribute to the prevention of associated symptoms. The main objective of this study is to evaluate the nutritional status and the prevalence of cardiometabolic risk factors in adults with CP and Gross Motor Function Classification System (GMFCS) levels between IV and V. *Materials and Methods:* A sample of 41 adults with CP and GMFCS levels from IV to V were studied. The variables used in the study were age, sex, weight, height, mean age, and GMFCS level range. To evaluate nutritional status, body mass index and the Mini Nutritional Assessment (MNA), a nutritional screening tool, were used. To assess cardiometabolic risk, data on obesity, central obesity, blood pressure, fasting plasma glucose, total cholesterol, high-density lipoprotein cholesterol, low-density lipoprotein cholesterol, and triglycerides were collected. *Results:* More than 80% of the population studied was malnourished or at risk of malnutrition, according to the MNA tool classification ranges, and around 35% of the studied population was within the underweight range. Regarding cardiometabolic risk factors, only one adult with CP was diagnosed with MS. *Conclusions:* The studied population of adults with CP and GMFCS levels between IV and V is not a population at risk of MS; however, the high prevalence of malnutrition, as well as some of the most prevalent cardiovascular risk factors, should be taken into consideration.

## 1. Introduction

Metabolic syndrome (MS) is becoming one of the main public health problems of the 21st century. It is a set of interrelated metabolic abnormalities that can cause the development of cardiovascular disease and type 2 diabetes mellitus. The risk factors of MS include elevated blood pressure; dyslipidemia, including increased triglycerides and lower high-density lipoprotein cholesterol (HDL-cholesterol); raised fasting plasma glucose; and central obesity [1].

The World Health Organization (WHO) defined the term MS for the first time in 1999; since then, various diagnostic criteria have been proposed by different organizations, and the most accepted are those developed by the European Group for the Study of Insulin Resistance, the International Diabetes Federation (IDF), and the Adult Treatment Panel III (ATP-III) of the National Cholesterol Education Program (NCEP) [2]. These organizations harmonized the criteria of MS components and unified the definition, creating a single set of cutoff points to be used for all parameters except waist circumference (WC; national or regional cutoff points for WC can be used) [1].

The prevalence of MS in Spain is around 40% [3]. Physical inactivity along with abdominal obesity and insulin resistance appear to be some of the most predominant underlying factors for MS [4]. People with a low level of physical activity or a chronic physical condition are at risk of developing MS. Cerebral palsy (CP) disease is a set of permanent disorders that affect the development of movement and posture, which limit general activity and, therefore, could contribute to the development of MS. 

Population-based studies report that the overall birth prevalence of CP is approximately 2 per 1000 live births [5]. In Spain, the estimated population with CP is around 120,000 people. Although this prevalence has been stable over the past few years, there is no report on the prevalence of CP in the adult population. No standard of follow-up exists for individuals with CP who live into middle and later adulthood, and little is known about long-term health trajectories among adults in this population. All the prevalence statistics for adults are usually extrapolated [6,7]. Although CP is considered a non-progressive disorder, at least a quarter of the adults with CP also experience a decline in mobility by the age of 40 years [8]. It has been evidenced that CP is associated with worsening mobility in young adulthood, accelerated loss of muscle mass, fatigue, progression of motor dysfunction, and reduced participation in physical activity [9]. In individuals with mild-to-moderate impairment, heart disease causes 15% of deaths [10]. As most adults with CP exhibit sedentary behavior [11], adequate nutrition can contribute to the prevention of symptoms associated with CP and, in turn, protect against the appearance of cardiometabolic risk factors. Although there is no cure for CP, the treatment and prevention of such symptoms can help improve the quality of life of people with CP. In addition, a healthy diet and an adequate percentage of body fat could reduce the risk of metabolic disease. Recent research on nutritional status in individuals with CP shows a high prevalence of undernutrition in this population [12,13,14]. Suboptimal nutrition has been detected in children with mild-to-severe CP and also in adults. Despite the little existing data on the nutritional status of aging adults with CP, severe Gross Motor Function Classification System (GMFCS) levels are related to worse nutritional status and higher prevalence of multimorbidity [15,16]. Therefore, the main objective of this study is to evaluate the nutritional status and prevalence of cardiometabolic risk factors in institutionalized adults with CP, particularly severe CP or CP with GMFCS levels between IV and V. 

## 2. Materials and Methods

### 2.1. Data Collection 

A descriptive and observational analytical study of adults with CP (*n* = 41), including 25 men and 16 women between 18 and 62 years of age, was developed. The participants were recruited from a national center that provides services to people with a disability in Spain. The inclusion criteria were as follows: adults, with CP, hospitalized in the studied center. The approval for this study was granted by the Infanta Elena CP Center in Alicante. The participants were informed of the testing procedures before informed written consent was obtained. In the case of participants with a mild-to-moderate intellectual disability, their caregivers also provided informed written consent. The ethical board approval number from Alicante University is (UA-2018-07-05).

### 2.2. Variables

The variables used in this study were sex, age (years), GMFCS levels [17], weight (kg), height (cm), WC (cm), systolic blood pressure (mmHg), diastolic blood pressure (mmHg), fasting plasma glucose (mg/dL), total serum cholesterol (mg/dL), serum HDL-cholesterol (mg/dL), serum low density lipoprotein-cholesterol (LDL-cholesterol) (mg/dL), and serum triglycerides (mg/dL).

Anthropometric data were measured in the participants. For non-ambulatory adults, stature was predicted from knee height [18]—the distance from the posterior surface of the thigh to the sole of the foot when the knee is bent at 90°. Knee height was measured using calipers. WC was measured on bare skin to the nearest 0.1 cm, midway between the lower rib margin and the iliac crest, at the end of a gentle expiration. WC was measured in ambulatory participants in the standing position and in non-ambulatory participants in the supine lying position. The mean of the two measurements was calculated. 

Blood pressure was measured in the right arm or the less-affected side. The participants were seated in a warm, quiet room, with the upper arm at heart level. The cuff was placed so that the lower edge was 3 cm above the elbow crease and the bladder was centered over the brachial artery. The arm circumference was also measured to allow the appropriate cuff size to be selected. Sequential same-arm measurements were independently recorded using an Omron 705IT test device, which has good validity for blood pressure measurement according to the British Hypertension Society criteria [19].

Institutionalized individuals are periodically evaluated for the clinical parameters included in this study by a systematic protocol. Therefore, to collect the clinical data associated with cardiometabolic risk factors, the institution archives were accessed, and the most recent data were collected to create an online database. 

To evaluate nutritional status, body mass index (BMI) was used, in addition to the Mini Nutritional Assessment (MNA), a nutritional screening tool [20]. BMI (kg/m2) was calculated from anthropometric data by trained personnel, and the categories were defined according to the WHO criteria [21]. The MNA is a test that results in the assignation of people into three groups: 12–14 points, normal nutritional status; 8–11 points, risk of malnutrition; and 0–7 points, malnutrition [20]. 

To assess cardiometabolic risk factors, data on obesity, central obesity, blood pressure, fasting plasma glucose, serum values of total cholesterol, HDL-cholesterol, LDL-cholesterol, and triglycerides were collected. Fasting blood samples were collected early in the morning. Extracted samples were analyzed according to the established protocol of the external laboratory with which the CP center collaborates. Based on the cardiovascular risk factors, MS was diagnosed according to the ATP-III [22] and IDF [23] criteria. According to the ATP-III criteria, adults with MS were defined as those who presented at least three of the following five symptoms: abdominal obesity, elevated triglycerides (or drug treatment for elevated triglycerides), reduced HDL-cholesterol (or drug treatment for reduced HDL-cholesterol), elevated blood pressure (or antihypertensive drug treatment), and elevated fasting plasma glucose (or type 2 diabetes mellitus or drug treatment of elevated glucose). As is mandatory for the IDF criteria, one of these symptoms must include abdominal obesity.

### 2.3. Statistical Analysis

The characteristics of the participants were described as the mean ± standard deviation (SD) or percentages. The online database was analyzed using SPSS Statistics software for Windows version 22.0 (IBM, Spain). Parametric or nonparametric statistics were applied depending on the goodness-of-fit for normal distribution as determined by the Shapiro–Wilk test and the equality of variances among groups as determined by Levene’s test. In addition, the effect of the variables analyzed on the cardiometabolic risk factors was assessed using Spearman’s rank test for correlations. Parametric quantitative data were analyzed by *t*-test. Analysis of nonparametric quantitative data was performed with Mann–Whitney’s *U* test. The statistical significance threshold was *p* < 0.05.

## 3. Results

The sociodemographic, anthropometric, and clinical characteristics of the participants with CP are presented in Table 1. All the subjects included were adults around 40 years of age, although the group of the men included a 62-year-old individual. For both genders, the mean values of BMI and WC were within the normal range. According to the GMFCS, only 3 individuals (two women and one man) were classified as level IV, and the rest of them were level V.

Regarding the clinical parameters, as can be observed in Table 1, mean values of systolic and diastolic blood pressure were within the normal range for both genders. The same result was observed for the values of fasting plasma glucose and serum triglycerides. No significant differences were found between men and women for these parameters. The mean values of total cholesterol for women were higher than for men (*p* = 0.038). This may be due to the high levels of LDL-cholesterol in this group. Although only four individuals included in the study had total cholesterol above the normal range, 16 (39%) had high levels of LDL-cholesterol, and 11 (27%) of them also had low HDL-cholesterol values.

Regarding the nutritional status assessment, the classification obtained with the BMI data is shown in Table 2. Although, in both genders, most of the individuals were of normal weight, around 35% of the studied population was within the underweight range, this being more common among the men. The frequency of obesity was less than 10% for both genders, but the overweight prevalence was notably higher in women (31.3%) than in men (8.0%). However, none of those differences in BMI classification were statistically significant.

The results of the MNA screening tool are shown in Table 3. More than 80% of the population studied was malnourished or at risk of malnutrition, according to the MNA tool classification ranges. About half of the individuals were classified as malnourished, but no significant differences were found between women and men according to the MNA results.

When correlations between cardiometabolic risk factors and nutritional status (by BMI and MNA) were studied, no significant differences were found. Only a positive correlation between WC and BMI was found (*r*^2^ = 0.806, *p* = 0.000). Additionally, no significant differences between sex and GMFCS levels were found.

Two different sets of criteria proposed for the clinical diagnosis of cardiometabolic risk have been used, ATP-III and IDF. The results obtained for the metabolic parameters measured are presented in Table 4. According to the ATP-III criteria, only one of the participants can be assessed as a 10-year (short-term) cardiometabolic risk. To be classified as a cardiometabolic risk, an individual must show at least three of the risk factors. In our study, for MS diagnosis, the results applying the two criteria proposed were the same. Only one man with three risk factors (WC, hyperglycemia, and hypertension drug treatment) was diagnosed with MS, including a high value of his WC, as is mandatory in the IDF criteria. This individual was also obese and was at risk of malnutrition (based on the MNA criteria). 

On the other hand, only one participant (a woman) had two risk factors simultaneously according to the ATP-III criteria. However, according to the IDF criteria, two women with two risk factors were found. Among the risk factors detected, the most prevalent were abdominal obesity and high fasting plasma glucose.

The results obtained show that 90% of the adults with obesity or who were overweight had one or more risk factors, and 70% of them were malnourished or at risk of malnutrition according to the MNA results. On the contrary, only one of the 14 underweight adults had one cardiometabolic risk factor.

## 4. Discussion

The aim of this study was to report the prevalence of malnutrition and cardiometabolic risk factors in institutionalized adults with CP and GMFCS levels between IV and V. The results indicate that the studied population of adults with severe CP showed a low prevalence of cardiometabolic risk factors. However, their nutritional status requires more attention. Most of the sample studied was malnourished or at risk of malnutrition according to the MNA results.

Differences in physical activity capacity among our sample were not assessed, because these individuals had very limited mobility. According to the GMFCS, level IV indicates self-mobility with a wheelchair with limitations, and level V includes individuals who must be transported in a manual wheelchair [6]. Although two different GMFCS levels were included in the study, only three of the participants belong to level IV, and their mobility does not differ significantly from the level V subjects.

Several studies showed that swallowing problems are significantly more common in individuals with severe CP. GMFCS levels between IV and V have been closely related to dysphagia in children [24]. Although the literature concerning adults with CP is scarce, adults with CP often present swallowing disorders and feeding difficulties [25]. Among the most common health problems in the population with CP are alterations of certain aspects of ingestion, such as salivation, swallowing, and aspiration. Digestion problems, such as gastro-esophageal reflux and delayed gastric emptying, are also common. These alterations are manifested in endocrinal, gastrointestinal, and absorption complications. Moreover, in children with CP posture problems, complications with respect to food intake have been described [26,27]. The results obtained regarding malnutrition in the present study could be the consequence of these motor and swallowing problems. For the diagnosis of malnutrition risk and underweight status, we used two different criteria: the MNA screening tool and BMI cutoffs, respectively. Although little research exists about the use of nutritional screening tools for adults with CP, the MNA test has been described as one of the reference methods used for institutionalized adults. It allows for a nutritional evaluation without the need for complementary tests [28,29]. Here, the MNA classification showed that almost half of the individuals were malnourished, and one-third of the sample was at risk of malnutrition. However, when applying BMI cutoffs, the prevalence of underweight individuals was about 35%. Some of the adults classified as undernourished by the MNA test could be included in other BMI subgroups. This difference may be due to the fact that the BMI cutoff values commonly used to diagnose obesity have high specificity but have low sensitivity to identify adiposity and do not discriminate between adipose tissue and muscle [30,31]. This can be a problem in the population with CP, for both adults and children, due to the risk of significant muscle atrophy and diminished bone density [10,30,32,33]. Moreover, the validity of BMI is highly suspected in individuals with CP [34]. Even though BMI is not the gold standard to determine nutritional status, it is the easiest and most frequently used to classified weight status in adults with CP [15,35,36,37]. Our BMI results showed a prevalence of obesity of 7.3%, which agrees with several studies on adults with CP from European populations [35,36]. Nevertheless, this value is lower than those found in American and Canadian populations with CP [15,38]. This difference leads us to believe that the origin of the sample could influence the obesity-related parameters, as it does for the WC cutoffs [35,39]. Another of the factors that may have an influence on the results regarding nutritional status, is the GMFCS level. Recent research highlights the recognized risk of undernutrition in adults with CP, especially in those who are severely impaired [16,40]. Moreover, the studies developed with children with CP support the relationship between undernutrition and severe CP [12,13,14].

Because BMI lacks the sensitivity to identify non-obese individuals with excess body fat [30], it is not the recommended tool for characterizing cardiometabolic health among adults with CP [37]. Although overweight status and obesity are associated with insulin resistance and MS, the presence of abdominal obesity is more highly correlated with metabolic risk factors than an elevated BMI. Therefore, the simple measurement of WC is recommended in order to identify abdominal adiposity and the body weight component of MS [35,39]. In our study, WC was measured and used to identify risk factors according to two different sets of criteria, the ATP-III and the IDF criteria. The former has a less restrictive criterion. However, the IDF criteria state that the criterion of abdominal obesity must be specified by nationality or ethnicity.

As the present study focused on Spanish adults with CP, European values of central obesity were used (≥94 cm in men, and ≥80 cm in women) [41]. Our results obtained using the criteria described above showed a prevalence of central obesity of around 20% in the population, which agrees with data obtained by Van der Slot et al. (2013), also in a European sample [36]. In contrast, a study on cardiometabolic risk factors in an Irish population with CP obtained higher values (36%) of central obesity, including adults with CP of all GMFCS levels [35]. These disparities may be due to the differences in GMFCS levels of the studied samples. According to the literature, GMFCS seems to be related with central obesity, and individuals with levels between IV and V show a lower prevalence of central obesity when compared with individuals with levels between I and III of this classification [35,37]. 

The ATP-III criteria for WC, also used in our work, were defined for Americans [15]. Our results showed a prevalence of central obesity of 9.8%, which can be considered a low value in comparison with the results obtained with the IDF criteria (adapted to a European population). In this context, a recent study developed in Canada found a prevalence of central obesity close to 30% [36]. Once more, this highlights the need to use criteria adapted to the general characteristics of each population.

Only one man with a high value (2.4%) of hypertriglyceridemia was found. The average value of the total sample was below the cutoff established for both criteria used. This result agrees with Ryan et al. (2014), who found similar values of serum triglycerides [11]. The same authors found similar mean values but a higher prevalence of hypertriglyceridemia in another study about cardiometabolic risk factors in adults with CP [35].

Around 27% of the sample presented low levels of HDL-cholesterol. Even though the average HDL-cholesterol level for women was higher than that for men, most of the adults with CP who presented this MS risk factor were women. In accordance with our results, McPhee et al. reported a prevalence of 24.2% [38]. However, other authors obtained lower prevalence [35,36]. Low values of HDL-cholesterol in our sample may contribute to the normal range of total cholesterol values found, which agrees with other studies developed in adults with CP [11,35,36]. However, the average LDL-cholesterol values in those same studies, as well as in the present one, exceed the recommended level. It should be emphasized that women had higher levels of LDL-cholesterol and total cholesterol than men [11,35,36].

The average fasting plasma glucose values obtained agree with those of the studies of Ryan et al. and Van der Slot et al., in all cases being under the limits established [11,36]. According to the APT-III and IDF criteria, four adults with CP had values of fasting plasma glucose above 100 mg/dL, and all of them were men. Hypertension presented a prevalence of 4.9%, and only one man presented this risk factor. The results in the literature about the prevalence of hyperglycemia and hypertensive blood pressure in adults with CP are very discordant, ranging from 0% to 51.5% and from 5.7% to 53.3%, respectively [35,36,38].

Despite the existence of a wide variety of cardiometabolic risk factors in our sample, only one of the individuals exhibited three risk factors simultaneously. Therefore, according to the criteria set for MS diagnosis, 2.4% of our adults with CP were diagnosed with MS. Data reported from similar studies on PC individuals reflect a prevalence of MS around 20% [11,35]. Although several authors have detected cardiovascular risk in this population, most of them did not classify the sample by MS diagnosis [33,35,36].

There are several limitations of the current study that point to potential areas for future research. Primarily, the data included in the present study were cross-sectional, and as a result, causation cannot be directly inferred. Secondly, the studied sample was relatively small and may have influenced the estimate of cardiometabolic risk. However, the results obtained can be considered of interest due to the lack of studies about nutritional and cardiometabolic risk among Spanish adults with CP. It should also be noted that the sample size is similar to those of other studies of adults with CP. A third limitation was that only narrow demographic information was collected. Future studies would benefit from the inclusion of a large sample size of participants from more diverse settings and regions in Spain.

Despite the small number of adults included in this study, from the results obtained, it can be concluded that the prevalence of MS risk factors in the studied population of adults with CP and GMFCS levels between IV and V is low. However, some of the most prevalent cardiovascular risk factors, such as low serum HDL-cholesterol level, should be taken into consideration. Moreover, the prevalence of malnutrition and underweight status in this sample is high, which is also a point to consider in people with CP. Recent studies on individuals with CP showed that data concerning children with CP are not applicable to adults [42]. Thus, population-based studies of adults with CP are needed, and our study can contribute to increased knowledge of chronic disease risk, malnutrition risk, and survival among adults with CP.

## Figures and Tables

**Table 1:** Participants’ characteristics, anthropometric measurements, and cardiometabolic outcomes in adults with cerebral palsy.

Characteristics ^1^	Total (*n* = 41)	Women (*n* = 16)	Men (*n* = 25)
Age (years)	40.5 ± 7.3	38.5 ± 5.1	41.0 ± 8.2
Age (years), range	30 – 62	30 – 39	32 – 62
Weight (kg)	49.5 ± 17.9	50.6 ± 12.6	50.4 ± 21.0
Height (cm) *	155 ± 0.8	150 ± 0.7	158 ± 0.7
Body Mass Index (kg/m^2^)	20.7 ± 7.4	22.1 ± 5.5	19.7 ± 8.4
Waist Circumference (cm)	78.0 ± 11.7	77.3 ± 12.0	78.4 ± 11.8
Systolic blood pressure (mmHg)	113.0 ± 11.2	110.0 ± 9.6	115.7 ± 12.0
Diastolic blood pressure (mmHg)	72.8 ± 15.8	75.0 ± 6.6	69.5 ± 19.5
Fasting plasma glucose (ml/dL)	84.7 ± 7.7	82.3 ± 4.2	86.3 ± 9.1
Serum total cholesterol (mg/dL) *	172.0 ± 27.9	183.0 ± 17.7	163.7 ± 31.7
Serum HDL-cholesterol (mg/dL)	48.0 ± 11.7	51.2 ± 8.2	45.0 ± 11.0
Serum LDL-cholesterol (mg/dL)	105.3 ± 30.6	116.3 ± 16.6	94.4 ± 37.5
Serum Triglycerides (mg/dL)	81.6 ± 33.2	76.6 ± 33.7	85.0 ± 33.3

^1^ With the exception of age range, results are expressed as mean ± standard deviation (SD). * *p* < 0.05. HDL: high-density lipoprotein, LDL: low-density lipoprotein.

**Table 2:** Prevalence of underweight, normal-weight, overweight, and obese adults with cerebral palsy, according to the body mass index (BMI) classification.

	Total (*n* = 41)	Women (*n* = 16)	Men (*n* = 25)
BMI classification	*n* (%)	*n* (%)	*n* (%)
Underweight	14 (34.1)	4 (25.0)	10 (40.0)
Normal weight	17 (41.5)	4 (37.5)	11 (44.0)
Overweight	7 (17.1)	5 (31.3)	2 (8.0)
Obese	3(7.3)	1 (6.3)	2 (8.0)

BMI: body mass index.

**Table 3:** Mini Nutritional Screening tool results in a population of adults with cerebral palsy.

	Total (*n* = 41)	Women (*n* = 16)	Men (*n* = 25)
Screening score	*n* (%)	*n* (%)	*n* (%)
Malnourished	20 (48.8)	7 (43.8)	13(52.0)
At risk of malnutrition	14 (34.1)	6 (37.6)	8 (32.0)
Normal nutritional status	7 (17.1)	3 (18.6)	4 (16.0)

**Table 4:** Results obtained by applying the criteria of the Adult Treatment Panel III (ATP-III) of the National Cholesterol Education Program and the International Diabetes Foundation (IDF) to diagnose metabolic syndrome.

		Total (*n* = 41)	Women (*n* = 16)	Men (*n* = 25)
		*n* (%)	*n* (%)	*n* (%)
Central obesity	(waist circumference >102 cm (male), >88 cm (female)) ^1^ (waist circumference ≥94 cm (male), ≥80 cm (female)) ^2^	4 (9.8) 8 (19.5)	3 (18.7) 5 (31.2)	1 (4.0) 3 (12.0)
Hypertriglyceridemia (triglycerides ≥150 mg/dL)		1 (2.4)	0 (0.0)	1 (4.0)
Low HDL-cholesterol (<40 mg/dL (male) and <50 mg/dL (female))		11 (26.8)	7 (43.8)	4 (16.0)
Fasting plasma glucose ≥100 mg/dL		4 (9.8)	0 (0.0)	4 (16.0)
Hypertension: blood pressure ≥130/85 mmHg		2 (4.9)	0 (0.0)	2 (8.0)

^1^ ATP-III criteria. ^2^ IDF criteria.
